# Breast cancer patients’ needs and perspectives on a one-on-one peer support program: quantitative and qualitative analyses

**DOI:** 10.1007/s00520-023-08009-6

**Published:** 2023-10-26

**Authors:** Britt AM Jansen, Claudia A Bargon, Tessa L Dinger, Myra van den Goor, Emily L Postma, Danny A Young-Afat, Helena M Verkooijen, Annemiek Doeksen

**Affiliations:** 1 Division of Imaging and Oncology, University Medical Centre (UMC) Utrecht, Heidelberglaan 100, 3584 CX Utrecht, The Netherlands; 2 Department of Surgery, St. Antonius Hospital, Soestwetering 1, 3543 AZ Utrecht, The Netherlands; 3 Department of Plastic, Reconstructive and Hand Surgery, St. Antonius Hospital, Soestwetering 1, 3543 AZ Utrecht, The Netherlands; 4 Q3 Performance, Company for Professional Physician Development, Den Bosch, The Netherlands; 5 Department of Plastic, Reconstructive and Hand Surgery, Amsterdam University Medical Centre, Amsterdam, The Netherlands; 6 Utrecht University (UU), Heidelberglaan 100, 3584 CX Utrecht, The Netherlands

**Keywords:** Breast cancer, Supportive care, Needs, One-on-one peer support, Peer support, Mental health

## Abstract

**Purpose:**

Although peer support programs as a health resource have become increasingly popular, only limited studies evaluated the added value of one-on-one peer support for breast cancer patients. This study aims to bridge the knowledge gap by focusing on two related research topics. First, we evaluated emotional well-being and (unmet) needs regarding supportive care. Second, we evaluated patients’ perspectives on their experiences after having one-on-one peer support.

**Methods:**

A quantitative analysis was conducted to provide insight in patients’ symptoms of anxiety and depression (HADS), quality of life (EORTC-QLQ-C30), and supportive care needs (CaSUN-questionnaire). Furthermore, approximately 1 year after the implementation of a one-on-one peer support program, focus groups were conducted to evaluate patients’ perspectives regarding one-on-one peer support.

**Results:**

Two hundred twenty-five of 537 patients diagnosed with breast cancer between 2019 and 2020 completed the questionnaires. Quantitative analysis showed increased symptoms of anxiety and depression among breast cancer patients and lower scores on all EORTC-QLQ-C30 domains compared to the Dutch normative population. Of all patients, 27.6% (95%CI = 0.22–0.34) reported to have unmet needs regarding emotional support and 23.1% (95%CI = 0.18–0.29) reported an unmet need to talk to someone who has experienced breast cancer. For the qualitative analysis, 19 breast cancer patients who were taking part in the one-on-one peer support program participated in three focus groups. Benefits, limitations, and wishes regarding the one-on-one peer support program were discussed.

**Conclusion:**

Breast cancer patients showed increased anxiety and depression and lower quality of life, physical, role, emotional, cognitive, and social functioning compared to the Dutch normative population. Almost one-third of breast cancer patients reported unmet needs regarding emotional support and a desire to talk to other breast cancer patients. These (unmet) needs can successfully be met by providing a low-threshold one-on-one peer support program.

**Supplementary information:**

The online version contains supplementary material available at 10.1007/s00520-023-08009-6.

## Introduction

Worldwide, breast cancer is the most frequently diagnosed malignancy in women [1]. Due to improvements in breast cancer survival rates, there is a growing focus on quality of life (QoL) of breast cancer patients and survivors [2]. Patients diagnosed with breast cancer are often overwhelmed by feelings of anxiety, uncertainty, and loneliness [3]. Both diagnosis and treatment can lead to impaired emotional, physical, and social functioning, all of which may affect patients’ perceived quality of life [4]. Depression and anxiety are frequently observed, which has been shown to increase pain perception [5], extended in-hospital stays [6], and results in poorer daily functioning [7, 8]. Moreover, over 40% of breast cancer patients experience long-term or permanently reduced workability [9].

Aiming to reduce these psychosocial side-effects, peer support is increasingly applied in the field of oncology. Peer support has been identified as an important form of social support for stressful experiences, such as undergoing treatment for breast cancer [10]. The essence of peer support is often beyond the scope of health professionals [11]. Unlike professional medical support, the fundamental premise of peer support is low-threshold, mutual support, based on common disease experience [12]. Being in touch with someone going through the same medical process may be valuable for selected patients, potentially improving their ability to cope with the diagnosis, provide insight in what they may expect during and after treatment, and how to cope with the impact and (adverse) effects of treatment. Previous literature confirmed that QoL-related outcomes were positively affected in women who attended Breast Cancer Self-Help Groups or other forms of peer support [4, 12]. A systematic review of peer support programs for patients with cancer recommended one-on-one face-to-face peer-support when considering peer support, as this effectively improves psychosocial functioning [13].

In the Netherlands, the Buddy House has been developed as a one-on-one peer support program and matches breast cancer patients with former or current patients based on individually chosen criteria. The Buddy House aims to improve QoL and psychosocial well-being of patients diagnosed with breast cancer.

Although peer support programs as a health resource have become increasingly popular around the world, only limited studies evaluated the added value of one-on-one peer support for breast cancer patients [14, 15]. Therefore, this study aims to bridge this knowledge gap by focusing on two related research topics. First, we evaluated emotional well-being and (unmet) needs regarding supportive care among breast cancer patients and compared it to the Dutch normative population. Second, we evaluated patients’ perspectives on their experiences after having one-on-one peer support.

## Methods

### Study design

This was a single-center study. In order to address both research topics, quantitative and qualitative data analysis was applied. To address our first research topic, i.e., provide insight in supportive care needs among breast cancer patients and evaluate their well-being, a quantitative assessment was conducted among breast cancer patients at the St. Antonius Hospital, the Netherlands. The second topic, i.e., patients’ experiences regarding one-on-one peer support, was addressed approximately 1 year after the introduction of the one-on-one peer support program, as part of the Buddy House, by using an interpretative phenomenological approach [16]. Focus groups were organized to stimulate collective interaction, and to emerge different thoughts, beliefs, and feelings. The COREQ (consolidated criteria for reporting qualitative research) checklist was used to assess methodological quality [17].

A waiver was provided by the Medical research Ethics Committee United (the Netherlands, Nieuwegein, W19.212). All participants provided informed consent for collection and use of data.

### Quantitative phase methodology

#### Participants

For the quantitative analysis of this study, all patients ≥ 18 years old, newly diagnosed with breast cancer in 2019 and 2020, were selected based on breast cancer diagnosis, provided by the Business Intelligence Department of the St. Antonius Hospital. Exclusion criteria included unknown email address, history of breast cancer, deceased by time of follow-up, no surgical treatment, treatment in another hospital, distant metastasis and not being able to understand and speak the Dutch language sufficiently. Patients eligible for inclusion were asked to participate in this study by email. Non-responders to each questionnaire were sent a one-time reminder after four weeks.

#### Data collection

Data collection was cross-sectional. All subjects who agreed to participate were sent questionnaires to evaluate patients’ symptoms of anxiety and depression (HADS-NL) [18], quality of life (EORTC-QLQ-C30)[19], and (un)met needs (CaSUN-NL) [20].

The HADS-NL was used to assess symptoms of anxiety and depression. This questionnaire includes seven items on symptoms of anxiety and seven on symptoms of depression. For both scales, a score of 8 or higher indicates clinically relevant symptoms of anxiety or depression.

The EORTC-QLQ-C30 was used to assess quality of life, patient satisfaction, and psychosocial well-being. A summary score for the subscales quality of life, physical, role, emotional, cognitive, and social functioning was calculated according to EORTC guidelines. Thresholds for clinical importance were used to support interpretation of summary scores [21].

The CaSUN-NL questionnaire includes 14 items and was used to assess the level of (un)met needs experienced by the participants. Domains of need were as follows: existential survivorship (i.e., cancer-related distress), comprehensive cancer care, information, quality of life, relationships, lifestyle, return to work, and positive change. Higher scores indicate greater (un)met needs.

Unadjusted normative data of the most recent HADS-NL (2017) and EORTC-QLQ-C30 (2018) questionnaires were obtained from the Patient Reported Outcomes Following Initial treatment and Long term Evaluation of Survivorship (PROFILES) registry [22]. PROFILES is a registry that examines the impact of cancer and its treatment on the physical and psychological well-being of a diverse group of cancer survivors. The availability of a control cohort of approximately 2000 persons from the general population who complete the same basic questionnaire annually provides the opportunity to estimate the unique impact of cancer, beyond that of normal aging and comorbidities.

Quantitative study data were collected and managed using REDCap electronic data capture tools [23].

#### Data analysis

Baseline demographics were summarized using frequencies and percentages. Continuous variables were presented as means with standard deviation (SD) or as median and interquartile range (IQR), as appropriate. Dichotomous and categorical data were presented as frequencies with percentages and 95% confidence intervals. Statistical analyses were conducted using IBM SPSS Statistics© version 26.

### Qualitative phase methodology

#### Participants and setting

All participants were members of the Buddy House, also called buddies, who completed the quantitative questionnaire and agreed to receive an invitation for focus group participation. We complied to the recommended number of four to ten participants per focus group [24]. Appendix I provides reflection on the various backgrounds of the authors, which is important in the context of the phenomenological approach.

#### Data collection

The research objective was explained in the focus group invitation and enlightened at the beginning of each focus group. All focus group meetings were supervised by an experienced focus group moderator who has experienced breast cancer herself (HW), ensuring empathy on this subject. The coordinating researcher (BAMJ) attended the meetings as an observer and summarizer. To stimulate participants would speak freely about potentially sensitive topics, medical staff was not invited. The moderator used a semi-structured interview guide addressing patients’ perspectives on their needs, expectations, and experiences. Notes were taken on a whiteboard, so participants could immediately provide feedback if the researcher (BAMJ) had misinterpreted subjects. Afterwards, a brief summary was sent to all participants to review findings of the meeting as a form of member check. All focus groups were audio-recorded with participants’ consent. Focus groups were conducted until thematic saturation was reached.

#### Data analysis

Audio-recordings were transcribed verbatim and anonymized by the researcher in the original language. Transcribed audio-records were analyzed using the ATLAS.ti 22© software program. Two independent researchers (BAMJ and TLD) used thematic content analysis with an inductive approach to identify common themes [25]. The authors first read the transcripts carefully to fully capture the context. Relevant units of the dataset were coded systemically to represent the meaning of each unit. After iteratively reviewing the data and codes, relating codes were grouped into categories. Further analysis was performed to generate category groups representing the relationship between the categories. The two authors independently performed the data analysis, after which they discussed codes, categories, and category groups in order to establish credibility in the interpretation of the data. In case of mismatching codes, discrepancies were resolved by discussion to reach consensus.

## Results

### Quantitative analysis

**Fig. 1:**
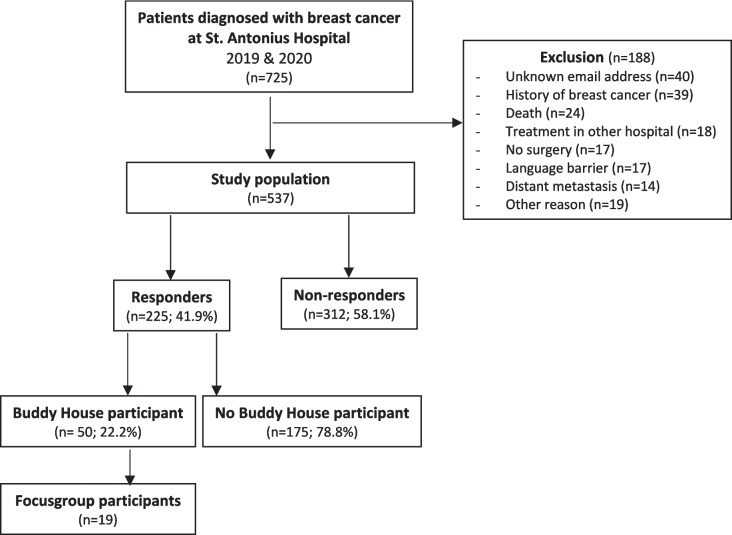
Flowchart of patient selection process

Patient selection resulted in the identification of 725 patients diagnosed with breast cancer in 2019 and 2020 (Fig. 1). After applying inclusion and exclusion criteria, 537 patients were invited to participate. A total of 225 patients (41.9%) responded to the invitation and completed the questionnaires (Table 1). Mean age was 58.1 years old (range 28–87), and mean interval between breast surgery and completion of the survey was 8 months (range 0–18). Most patients and survivors (63.6%) were married and had at least one child (81.3%). Half of the patients (49.3%) received conventional breast conserving surgery; 102 patients (45.3%) were treated with chemotherapy and 112 (49.8%) with hormone therapy.

**Table 1:** Baseline patient and treatment characteristics of responders (*n* = 225)

	Study population	
Patient characteristics		
Age in years, mean (range)	58	(28–87)
Years of education, mean (range)	16	(4–25)
Marital status, no. (%)		
Married	143	(63.6)
Unmarried	41	(18.2)
No partner	19	(8.4)
Widow	17	(7.6)
Other	5	(2.2)
Having ≥ 1 kids, no. (%)		
Yes	183	(81.3)
No	37	(16.4)
Employment status, no. (%)		
Full-time job	45	(20.0)
Part-time job	80	(35.6)
Unable to work	17	(7.6)
Unemployed	3	(1.3)
Other	82	(35.5)
Treatment characteristics		
Type of surgery, no. (%)		
Conventional or oncoplastic breast conserving surgery	143	(63.6)
Mastectomy with breast reconstruction	44	(19.6)
Mastectomy without breast reconstruction	28	(12.4)
Missing	10	(4.4)
Axillary treatment, no. (%)		
Sentinel node procedure	178	(79.1)
Axillary lymph node dissection	7	(3.1)
No axillary surgery	27	(12.0)
Missing	13	(5.8)
(Neo) adjuvant treatment, no. (%)		
Radiation therapy		
Yes	164	(72.9)
No	47	(20.9)
Missing	14	(6.2)
Chemotherapy		
Yes	102	(45.3)
No	110	(48.9)
Missing	13	(5.8)
Hormone therapy		
Yes	112	(49.8)
No	99	(44.0)
Missing	14	(6.2)
Time since breast cancer surgery in months, mean (range)	8.6	(0–18)
Missing, no. (%)	13	(5.8)

#### Symptoms of anxiety and depression

In total, 24.9% of all participants (95%CI = 0.20–0.31) showed clinically relevant symptoms of anxiety and 16.4% (95%CI = 0.12–0.22) symptoms of depression (Table 2). In the Dutch normative population, these proportions were 15.5% (95%CI = 0.14–0.17) and 13.2% (95%CI = 0.12–0.14), respectively [22].

**Table 2:** Physical and psychosocial wellbeing as measured by the HADS and the EORTC-QLQ-C30 questionnaires of the study population (*n* = 225) compared to the Dutch normative population (*n* = 3233 for HADS and *n* = 2521 for EORTC-QLQ-C30)

		Study population				Dutch normative population^a^			
HADS^b^		No.	%	95% CI		No.	%	95% CI	
Anxiety		56	24.9	0.20–0.31		502	15.5	0.14–0.17	
Depression		37	16.4	0.12–0.22		427	13.2	0.12–0.14	

EORTC-QLQ-C30^c^	TCI^d^	Mean	SD	95% CI	***n*** (%) of patients < TCI	Mean	SD	95% CI	***n*** (%) of patients < TCI
Physical functioning	83	83.5	19.1	81.0–86.0	83 (36.9)	91.0	15.0	90.3–91.5	470 (18.5)
Role functioning	58	76.5	27.8	72.8–80.1	45 (20)	89.6	20.7	88.8–90.4	218 (8.6)
Emotional functioning	71	78.4	23.4	75.3–81.5	66 (29.3)	85.9	19.2	85.1–86.6	477 (18.8)
Cognitive functioning	75	75.6	26.6	72.1–79.1	95 (42.2)	91.3	15.9	90.7–92.0	300 (11.8)
Social functioning	58	82.2	25.2	78.9–85.5	34 (15.1)	93.0	16.9	92.4–93. 7	135 (5.3)
Quality of life	-	73.1	20.1	70.4–75.7	-	76.3	18.3	75.6–77.1	-

Abbreviations: CI, confidence interval; EORTC, European Organization for Research and Treatment of Cancer; HADS, Hospital Anxiety and Depression Score; TCI, threshold for clinical importance

^a^Most recent representative PROs of the Dutch normative population from 2017 (HADS) and 2018 (EORTC-QLQ-C30)

^b^Number of patients having clinically relevant symptoms of anxiety or depression, based on total HADS scores of 8 or higher[18]

^c^EORTC-QLQ-C30 scores range from 0 to 100. Higher scores represent better outcomes[19]

^d^Number of patients with clinically important problems according to EORTC-QLQ0C30 threshold values reported by Giesinger et al.[21]

#### Quality of life core questionnaire

On the EORTC-QLQ-C30 questionnaire, all mean scores were lower than scores as measured in the Dutch normative population (Table 2) [22]. Interpretation of scores by means of clinical importance showed that 36.9% (*n* = 83; 95%CI = 0.31–0.43) scored lower than the threshold for clinical importance on the physical functioning scale, 20% (*n* = 45; 95%CI = 0.15–0.26) on the role functioning scale, 29.3% (*n* = 66; 95%CI = 0.23–0.36) on the emotional functioning scale, 42.2% (*n* = 95; 95%CI = 0.36–0.49) on the cognitive functioning scale, and 15% (*n* = 34; 95%CI = 0.11–0.20) on the social functioning scale [21].

#### Supportive care needs

Almost half of the participants (48.4%; 95%CI = 0.42–0.55) reported at least one unmet need in one of the five main domains, i.e., existential survivorship, comprehensive cancer care, information, quality of life, and relationships. The ten most frequently reported total, met, and unmet needs are listed in Table 3. In 27.6% of patients (95%CI = 0.22–0.34), unmet needs on emotional support were reported, and 23.1% (95%CI = 0.18–0.29) reported the unmet need to talk to someone who has experienced the same cancer.

**Table 3:** Top 10 unmet, met and total needs according to CASun questionnaire (*n* = 225)

		Number	Percent
Top 10 unmet needs			
1	Emotional support for me	62	27.6
2	Manage side effects	60	26.7
3	Concerns about cancer coming back	55	24.4
4	Talk to others	52	23.1
5	Survivor expectations	50	22.2
6	Reduce stress in my life	49	21.8
7	Acknowledging the impact	48	21.3
8	Changes to quality of life	44	19.6
9	Changes to my body	39	17.3
10	Move on with my life	39	17.3

Top 10 met needs			
1	Up to date information	71	31.6
2	Best medical care	60	26.7
3	Understandable information	52	23.1
4	Manage health with team	46	20.4
5	Doctor talk to each other	44	19.6
6	Information for others	41	18.2
7	Manage side effects	39	17.3
8	Emotional support for me	36	16.0
9	Talk to others	28	12.4
10	Reduce stress in my life	26	11.6

Top 10 total needs			
1	Up to date information	109	48.4
2	Manage side effects	99	44.0
3	Emotional support for me	98	43.6
4	Best medical care	93	41.4
5	Doctor talk to each other	82	36.5
6	Manage health with team	81	36.0
7	Talk to others	80	35.5
8	Reduce stress in my life	75	33.4
9	Concerns about cancer coming back	74	32.8
10	Understandable information	71	31.5

Abbreviations: CaSUN, cancer survivors’ unmet needs

### Qualitative analysis

In total, 19 women participated in three focus groups of the duration of one hour each (Supplementary Table 1). The mean age of participants was 50.5 years (SD = 11.4). Most women were married (*n* = 14, 73.7%) and currently employed (21.1% full-time, 47.4% part-time).

Qualitative data analysis resulted in the identification of 28 codes, 11 categories, and 3 categorical groups (Supplementary Table 2). The 11 categories were classified by the nature of their content into three categorical groups; benefits, limitations, and wishes regarding one-on-one peer support programs.

#### Benefits of one-on-one peer support

##### Mental support

“It was of great added value to me that I could share my worries and I no longer felt alone. Therefore, I felt less stress and I was able to continue the process and treatment. It truly contributed to my recovery process, mainly the mental recovery process.”

Patients often felt lonely in their medical process. Having a “buddy” (i.e., one-on-one peer support) brought comfort and confidence to patients and they felt it improved their mental well-being. Recognition among patients made it easy to talk to someone without further explanation and created an environment in which anything could be said. The ability to share concerns with a peer was seen as a stress relief and took away feelings of being lonely. Patients preferred discussing this emotional part of the medical process with peers rather than with medical staff. Participants also mentioned that having, or being a “buddy” to someone else, helped in their psychological recovery process.

##### Easily accessible

“I found it very difficult to ask for help, especially during the COVID-19 situation. Therefore, I was looking for a more accessible approach than making an appointment in the hospital, as I felt that a lot of people there needed it more than I did.”

Participants often felt uncomfortable asking for any kind of support from their doctors, nurses or loved ones. A low-threshold approach to a one-on-one peer program made it easier for participants to ask for support as they were all in the program for the same reason. There were no perceived limitations to when or where to join the peer program, which made the program accessible and tailored to patients’ needs.

##### Practical support

“The moment I experienced side effects, I asked my buddy if she had experienced the same. She did, and advised me to get a certain over-the-counter medicine. It is easier to ask your buddy than calling the specialized nurse again.”

Participants mentioned several advantages of receiving practical support by their buddies. Some advice, including non-medical advice, was not always mentioned by doctors or nurses. For example, for patients struggling with a physical disability after breast cancer surgery, it was considered to be useful to get some practical tips on how to facilitate daily activities at home. Also, personal experiences with different types of sports bras, chemotherapy hats or even surgical treatment options were commonly shared among buddies. Participants mentioned that some easy medical questions could be answered by buddies, resulting in less demand for medical consults.

##### Aftercare

“After I completed my treatment I noticed that everyone thought the breast cancer was past tense, but to me it felt like it only just began. But I only realize that now, two years later. At that time I thought: come on, let’s finish this treatment quickly. But you need a buddy to tell you to slow down.”

Participants reported that they found it difficult to adjust to life after breast cancer treatment had finished. When regular hospital visits became past tense, it felt like leaving behind a safe and familiar environment. According to participants, in this vulnerable and for some participants even the most difficult period, they wanted some extra guidance and attention. For that reason, many participants appreciated having access to the one-on-one peer support program.

##### Personal matching

“The way the connections are made are very personal, so the matches being made are very refined.”

Participants appreciate the personalized aspect of the matching process. Matches were mostly based on tumor and treatment characteristics, so patients could easily share experiences. For example, going through the same chemotherapy treatment at the same time while having the possibility to talk each other through it, made the treatment process more bearable. This also applied to patients who had already completed treatment that were supporting patients currently undergoing treatment.

Beneficial initiatives specific for the Buddy House program are reported in Appendix II.

#### Limitations of one-on-one peer support

##### Matching process challenges

“I had so many questions about the DIEP flap reconstruction, and that was our connection. But the match was only based on thetreatment and had nothing to do with a personal match.”

In case matches were based on tumor and treatment characteristics, the personal match was sometimes perceived as less successful. This was experienced as a limitation of the matching process by some, although it was still considered useful and pleasant to discuss specific treatment related topics. On the other hand, some treatment pathways might segregate over time, resulting in loss of interface.

##### Dealing with responsibility

“Sometimes I realize I’m not a therapist. But what do you do when someone’s concerningly devastated?”

Some participants struggled finding a balance between delivering support on one hand, while setting personal boundaries on the other. They have not been professionally trained to fulfill the role as a professional (mental) health provider.

##### Lack of clear expectations

“How do you know what to expect? Both for being a buddy as for having a buddy.”

Participants mentioned to sometimes lack clear expectations. There was some uncertainty regarding the preferred way of communication among buddies. Some buddies preferred to have digital contact through online chat only, while others favored face-to-face meetings. Participants also had different expectations on which topics could be discussed with buddies. For example, for some buddies talking about the breast cancer treatment is what they expect from a buddy, while others are looking for someone to share more personal and emotional details. Participants could also have preliminary incorrect expectations regarding the meaning of being someone’s buddy.

#### Wishes

##### Evaluation moment

“When a patient tells something that worries or touches you, there should be a possibility to ask for supervision.”

Participants would like the option to easily provide feedback to the Buddy House. For example, in case of a mismatch, a buddy wants to have the option to report when a patients’ personal story has too much impact, as this could negatively affect the mental status of another. The Buddy House could prevent buddies from being affected by someone’s story by inserting a moment of feedback after some weeks of contact.

##### Visible profile

“It could be helpful to show patients’ needs. For example, one person wants to talk about emotions, while someone else wants to ask practical questions regarding the operation.”

Several participants mentioned that the Buddy House could be improved by creating a buddy profile in which personal characteristics and preferences could be visualized. Providing visible communication preferences facilitates to meet each other’s expectations and needs.

## Discussion

This study evaluated patients’ needs and perspectives regarding a one-on-one peer support program for breast cancer patients. Our quantitative results showed that a considerable number of patients experienced more symptoms of anxiety and depression in comparison to the normative population (24.5% vs. 15.5% and 16.4% vs. 13.2%, respectively) [22]. Breast cancer patients reported lower quality of life, physical, role, emotional, cognitive, and social functioning than the Dutch normative population as measured by the EORTC-QLQ-C30. In total, 58.2% of all patients reported clinically important problems on at least one of the EORTC-QLQ-C30 functioning scales [21]. Additionally, a concerning proportion of participants (27.6%) reported to have unmet emotional support needs, and the need to talk to others who experienced cancer (23.1%). These findings underline the importance of psychosocial support for patients (being) treated for breast cancer. Our qualitative findings regarding one-on-one peer support showed benefits such as easily accessible, mental support, aftercare, practical support, and personal matching. Limitations included matching process challenges, dealing with the responsibility of being a buddy and unclear expectations. Patients suggested evaluation during peer support and provision of a visible profile.

Peer support programs are increasingly used to provide psychosocial support. The results of a systematic review by Hu et al. indicated that one-on-one peer support significantly improves negative emotions among breast cancer patients [26]. Our results provide deeper insights and showed that having a buddy even contributed to the emotional recovery process. These findings strongly underscore the benefits of one-on-one peer support on well-being of breast cancer patients.

Moreover, participants experienced added value of peer support regarding the choices they were facing. By personal match-making, women could easily gain information on personal experiences about surgical treatment options from buddies, adding value to their decision-making process. Shared decision making is currently the preferred model for making decisions in healthcare and encourages that patients have their say in selecting a treatment [27, 28]. However, previous literature suggests that patients may not feel proficient to participate due to lack of information or by being intimidated by the decision-making process in a vulnerable, often overwhelming phase after being confronted with the diagnosis of breast cancer [29, 30]. Our findings indicate that one-on-one peer support could play an empowering role in this process.

When using experiential expertise as an aid resource, additional training could be recommended [31]. The peer support system positions the peer, also a (former) patient, as an aid resource. This is likely to result into less demand for medical consults, thereby decreasing pressure on the currently overloaded healthcare system. However, only patients’ experiential knowledge is insufficient to call it expertise. Nevertheless, as the essence of peer support is offering experiential expertise, a peers’ role as an intermediary is thought to be a great additional benefit to (former) breast cancer patients and the medical system [31].

In addition to the psychosocial benefits of one-on-one peer support on individual level, a recent analysis even showed additional economic and social value of peer support programs. The social return on investment (SROI) analysis evaluated the impact of peer support on patients, health insurance, employers, municipalities and sponsors [32]. The results of the SROI cost-benefit analysis indicated that every euro invested in (any kind of) peer support yields a social value of €4,50 [32]. Most important value drivers were increased QoL, improved financial position, less absenteeism, and healthcare cost reduction.

This study had some strengths and limitations. The generalizability of these results might be subject to limitations such as selective (non-)response (e.g., if only patients with a positive experience participated in the focus groups, and none of those with less positive experiences). Although it remains unclear whether our results can be generalized to other patient groups (as they might have different supportive care needs), a randomized controlled trial (RCT) of Weber et al. is in accordance with our findings, as they showed significant positive changes on depression rates when investigating the effect of one-on-one support for men with prostate cancer [33]. An important strength of this study is its exploratory character, providing valuable insights in both patients’ unmet needs and how to overcome some of these unmet needs by one-on-one peer support. Particularly our qualitative approach gathered information on benefits as well clear directions for improvement of one-on-one peer support. The absence of medical staff during the focus groups created a safe environment motivating patients to openly give their opinion.

## Conclusion

Our quantitative results showed increased anxiety and depression among breast cancer patients and lower quality of life, physical, role, emotional, cognitive, and social functioning compared to the Dutch normative population. Patients mainly reported (unmet) needs on emotional support and (unmet) needs to talk to someone who has experienced the same cancer. Given the increasing number of breast cancer patients and survivors, and the load of (un)met social and emotional support needs, there is growing interest for peer support programs. This study identified benefits, limitations, and wishes regarding one-on-one peer support. Participants particularly emphasized the added value of a one-on-one peer support program for both during and after breast cancer treatment. These results may contribute to the development of new and improvement of existing one-on-one peer support programs.

### Supplementary information

Below is the link to the electronic supplementary material.

Supplementary file1 (DOCX 36 KB)

## Data Availability

Data is available upon request from the corresponding author.

## Abbreviations

BCBH: Breast Cancer Buddy House
CaSUN: Cancer Survivors’ Unmet Needs
COREQ: Consolidated criteria for Reporting Qualitative research
DIEP: Deep inferior epigastric perforator
EORTC: European Organization for Research and Treatment of Cancer
HADS: Hospital Anxiety and Depression Scale
QoL: Quality of life

## References

[1] Siegel RL, Miller KD, Fuchs HE, Jemal A (2022). Cancer Statistics, 2022. CA Cancer J Clin.

[2] Salakari M, Pylkkänen L, Sillanmäki L (2017). Social support and breast cancer: a comparatory study of breast cancer survivors, women with mental depression, women with hypertension and healthy female controls. The Breast..

[3] Johansson M, Rydén A, Finizia C (2011). Mental adjustment to cancer and its relation to anxiety, depression, HRQL and survival in patients with laryngeal cancer - a longitudinal study. BMC Cancer..

[4] Sowa M, Głowacka-Mrotek I, Monastyrska E (2018). Assessment of quality of life in women five years after breast cancer surgery, members of Breast Cancer Self-Help Groups - non-randomized, cross-sectional study. Contemp Oncol (Pozn)..

[5] Baqutayan SMS (2012). The effect of anxiety on breast cancer patients. India J Psychol Med.

[6] Nipp RD, El-Jawahri A, Moran SM (2017). The relationship between physical and psychological symptoms and health care utilization in hospitalized patients with advanced cancer. Cancer..

[7] Aryankhesal A, Ghashghaee A, Sardari E (2022). Prevalence of depression in patients with cancer in Iran: a systematic review and meta-analysis. BMJ Support Palliat Care.

[8] Barrett-Bernstein M, Carli F, Gamsa A (2019). Depression and functional status in colorectal cancer patients awaiting surgery: impact of a multimodal prehabilitation program. Health Psychology..

[9] Hauglann B, Benth JŠ, Fosså SD, Dahl AA (2012). A cohort study of permanently reduced work ability in breast cancer patients. J Cancer Survivor.

[10] Giese-Davis J, Bliss-Isberg C, Wittenberg L (2016). Peer-counseling for women newly diagnosed with breast cancer: a randomized community/research collaboration trial. Cancer.

[11] Mastrovito R, Moynihan R, Parsonnet L (1989) Self-help and mutual support programs. Handbook of psycho-oncology: psychological care of the patient with cancer New York: University Press, Oxford, pp 502–507

[12] Legg M, Hyde MK, Occhipinti S, Youl PH, Dunn J, Chambers SK (2019). A prospective and population-based inquiry on the use and acceptability of peer support for women newly diagnosed with breast cancer. Support Care Cancer.

[13] Hoey LM, Ieropoli SC, White VM, Jefford M (2008). Systematic review of peer-support programs for people with cancer. Patient Educ Couns.

[14] Rankin N, Williams P, Davis C, Girgis A (2004). The use and acceptability of a one-on-one peer support program for Australian women with early breast cancer. Patient Educ Couns.

[15] Ashbury FD, Cameron C, Mercer SL, Fitch M, Nielsen E (1998). One-on-one peer support and quality of life for breast cancer patients. Patient Educ Couns.

[16] Finlay L, Ballinger C (2006) Qualitative research for allied health professionals: challenging choices. John Wiley & Sons

[17] Tong A, Sainsbury P, Craig J (2007). Consolidated criteria for reporting qualitative research (COREQ): a 32-item checklist for interviews and focus groups. Int J Qual Health Care.

[18] Spinhoven P, Ormel J, Sloekers P, Kempen G, Speckens AE, van Hemert AM (1997). A validation study of the Hospital Anxiety and Depression Scale (HADS) in different groups of Dutch subjects. Psychological medicine..

[19] Fayers P, Aaronson NK, Bjordal K, Sullivan M (1995) EORTC QLQ–C30 scoring manual. Europ Org Res Treat Cancer

[20] Keeman M, Bolman C, Mesters I, Willems R, Kanera I, Lechner L (2018). Psychometric properties of the Dutch extended Cancer Survivors’ Unmet Needs measure (CaSUN-NL). Europ J Cancer Care.

[21] Giesinger JM, Loth FLC, Aaronson NK (2020). Thresholds for clinical importance were established to improve interpretation of the EORTC QLQ-C30 in clinical practice and research. J Clin Epidemiol..

[22] Mols F, Husson O, Oudejans M, Vlooswijk C, Horevoorts N, van de Poll-Franse LV (2018). Reference data of the EORTC QLQ-C30 questionnaire: five consecutive annual assessments of approximately 2000 representative Dutch men and women. Acta Oncol..

[23] Harris PA, Taylor R, Thielke R, Payne J, Gonzalez N, Conde JG (2009). Research electronic data capture (REDCap)—a metadata-driven methodology and workflow process for providing translational research informatics support. J Biomed Inform.

[24] Stake RE (2006) Multiple case study analysis. New York, NY: Guilford. ISBN: 1462512402.

[25] Braun V, Clarke V (2006). Using thematic analysis in psychology. Qual Res Psychol.

[26] Hu J, Wang X, Guo S (2019). Peer support interventions for breast cancer patients: a systematic review. Breast Cancer Res Treat.

[27] Kehl KL, Landrum MB, Arora NK et al (2015) Association of actual and preferred decision roles with patient-reported quality of care: shared decision making in cancer care. JAMA Oncol 1(1):50–58. 10.1001/jamaoncol.2014.112 10.1001/jamaoncol.2014.112 PMC4937185 26182303

[28] Stacey D, Légaré F, Lewis K et al (2017) Decision aids for people facing health treatment or screening decisions. Cochrane Database Syst Rev (4). 10.1002/14651858.CD001431.pub5 10.1002/14651858.CD001431.pub5 PMC6478132 28402085

[29] Berlin NL, Tandon VJ, Hawley ST (2019). Feasibility and efficacy of decision aids to improve decision making for postmastectomy breast reconstruction: a systematic review and meta-analysis. Med Decis Making..

[30] Waljee JF, Rogers MAM, Alderman AK (2007). Decision aids and breast cancer: do they influence choice for surgery and knowledge of treatment options?. J Clin Oncol.

[31] Helder AA. Internal publication PGO support, Utrecht Web site. https://helder-advies.nl/wp-content/uploads/2020/04/Lotgenotencontact-en-ervaringsdeskundigheid.pdf. Published 2021. Accessed

[32] Ploeg M, Ketelaar P. Social return on investment (SROI) analysis, PGO support. March 2021. www.pgosupport.nl/dossiers/patientenorganisaties/lotgenotencontact/maatschappelijke-waarde-sroi

[33] Weber BA, Roberts BL, Resnick M (2004). The effect of dyadic intervention on self-efficacy, social support, and depression for men with prostate cancer. Psycho-Oncol.

