# Supplementary material for: Breast cancer patients’ needs and perspectives on a one-on-one peer support program: quantitative and qualitative analyses
Source: Support Care Cancer. 2023 Oct 26;31(11):656. doi: 10.1007/s00520-023-08009-6 (PMC10602952; doi:10.1007/s00520-023-08009-6)
Supplement: Supplementary file 1 — Supplementary file1 (DOCX 36 KB) [file 520_2023_8009_MOESM1_ESM.docx]

**Supplementary materials - Index**

**Supplementary Tables**

Table S1: Demographics of focus group participants p. 1

Table S2: Representative quotes of focus group participants p. 2

**Appendices**

Appendix I: Reflexitivity p. 4

Appendix II: Beneficial initiatives specific for the Buddy House program p. 5**Supplementary Tables**

| **Table S1.** Demographics of focus group participants (n = 19). | | |
| --- | --- | --- |
|  | **Study population** | |
| **Patient characteristics** |  |  |
| Age in years, mean (range) | 50.5 | (30.2 - 71.7) |
| Years of education, mean (range) | 16.3 | (10.0 - 22.0) |
| Marital status, No (%) |  |  |
| Married | 14 | (73.7) |
| Unmarried | 3 | (15.8) |
| No partner | 1 | (5.3) |
| Widow | 1 | (5.3) |
| Having ≥1 kids, No (%) |  |  |
| Yes | 17 | (89.5) |
| No | 2 | (10.5) |
| Employment status, No (%) |  |  |
| Fulltime job | 4 | (21.1) |
| Partime job | 9 | (47.4) |
| Unable to work | 2 | (10.5) |
| Other | 4 | (21.0) |
| **Treatment characteristics** |  |  |
| Type of surgery, No (%) |  |  |
| Conventional breast conserving surgery | 7 | (38.9) |
| Oncoplastic breast conserving surgery | 1 | (5.6) |
| Mastectomy | 10 | (52.6) |
| Axillary treatment, No (%) |  |  |
| Sentinel node procedure | 18 | (94.7) |
| No surgery | 1 | (5.3) |
| (Neo) adjuvant treatment, No (%) |  |  |
| *Radiation therapy* |  |  |
| Yes | 16 | (84.2) |
| No | 3 | (15.8) |
| *Chemotherapy* |  |  |
| Yes | 10 | (52.6) |
| No | 9 | (47.4) |
| *Hormone therapy* |  |  |
| Yes | 13 | (68.4) |
| No | 6 | (31.6) |
|  | | |

| **Table S2.** Representative quotes of focus group participants (n = 19) classified into 28 codes, 11 categories and 3 category groups. | | | |
| --- | --- | --- | --- |
| **Categorical groups** | **Category** | **codes** | **Examples of content** |
| Benefits of one-on-one peer support | Easily accessible | More accessible than medical care | “I find it very difficult to ask for help, especially during the COVID-19 situation. Therefore I was looking for a more accessible approach than making an appointment in the hospital, as I felt that a lot of people there needed it more than I did.” |
|  |  | Easy approach | “I immediately got in touch that week. *Buddy A* called me immediately and I really liked that. That first step is just really difficult, and it was so nice that she called me or sent me a text message.” |
|  |  | According to own needs | “If I were your buddy, and you said to me: ‘I have so much trouble talking about it.’ I would start talking about other things, and we’ll get there eventually. Or maybe not, thats up to you then.” |
|  |  |  | “I really appreciate that I can just join whenever I need it. For example the walking groups. I started my re-integration process and started working again. But I know that if I want to, and I have a morning off, I can easily join the walking group.” |
|  | Mental support | Want to talk about emotions | “The whole medical process I discussed with my doctor and nurses. But the emotional part and the after care, I discussed with my buddy and other peer supporters in the walking groups.” |
|  |  | Reassuring each other | “Sometimes when we worry about things, we first reassure each other like: ‘it doesn’t have to be ‘this’ or ‘that’, but make sure you just let it check.’ You don’t follow your own advice, but only someone else’s.” |
|  |  | Emotional processing | “It was a great added value to me that I could share my worries and felt no longer alone. Therefore, I felt less stress and I was able to continue on the process and treatment and recover better. It truly contributed to my recovery process, mainly the mental recovery process.” |
|  |  |  | “By helping someone else, you emotionally process yourself as well.” |
|  |  | Need to tell their story | “I think it is very helpful for a buddy to have someone to be able to share your story without limitations, because a lot of people feel bothered by family, husband or children and don’t want to share the story over and over again.” |
|  |  | Recognition | “Great actually, to talk with a peer sufferer you don’t have to explain everything. That’s different when someone says: ‘you look good, why don’t you start working again?’ I really appreciated that.” |
|  |  |  | “It just feels good to hear from people who have experienced it and know how tough it is with children, work and socially, and that it is not just not that easy. Then it is great to share with someone who has experienced the same.” |
|  | Aftercare | Re-integration process | “After I completed my treatment I noticed that everyone thought the breast cancer was past tense, but to me it all just began. I only see that now, two years later. At that time I thought: come on, let’s do this quick. But you need a buddy to tell you to slow down.” |
|  | Practical support | Additional information not provided by medical staff | “Arrange homecare in time, make sure you have an adjustable bed. Because when everyone is off to school or work, how do you get out of bed? Make sure they prepare your breakfast, because when you get downstairs, you won’t be able to carry a kettle. That’s wat my buddy told me. No one told me about that in the hospital.” |
|  |  |  | “My buddy said: bring an extension cord, because you will be in bed for five days and you cannot get out of bed easily or reach out to anything. The nurse asked me what all that stuff was. I told her: my buddy told me this. That was very practical.” |
|  |  | Decision making assistance | “Do I want a prosthesis or not? What kind of prosthesis? Remove both breasts or remove only one? That kind of practical questions. For my buddy it was three years ago that she was in the same position as I am now. She was very good in talking about it. She had exactly the same considerations, and the same lists with pros and cons. Then I thought, we have so many similarities, and she made that choice, then I’ll go for it. So those were practical considerations.” |
|  |  | Less need for contact with doctor or nurses | “The moment I experienced side effects, I asked my buddy if she had experienced the same. She did and advised me to get a certain over-the-counter medicine. It is easier to ask your buddy than calling the specialized nurse again.” |
|  | Personal matching | Shared experiences | "My buddy gave me some really good tips, because she had the same chemotherapy. She told me what to expect, for example what side effects could occur. Those are really practical things. I was glad to know that in advance. But then it really comes down to a good match. That means the same chemotherapy or the same operation. Because in case that does not match, it is hard to support each other." |
|  |  |  | “The way the connections are made are very personal, so the matches being made are very refined.” |
|  |  | Easy understandeable | "Sometimes I try to let her see things from another point of view. That is easy if you have experienced the same. So the personal match making is really nice." |
|  | Beneficial initiatives specific for the Buddy House program | Walking groups | "I did not exactly know what I wanted or what I needed the most. So to me the walking groups were really pleasant, because I could just join in. Here I met four ladies with whom I still have a lot of contact and that is really nice." |
|  |  |  | "It is easy talking while you are walking and you meet different people." |
|  |  | Webinars | "On the website of the Buddy House you can replay the Webinars. I am not an evening person, so for me that is really nice." |
|  |  | Walk-ins | "Also the walk-ins were really helpful, because to me it was like; You have this imagination of having cancer, mainly old people. Then I saw *buddy C* sitting there, and all the other ladies who survived breast cancer. That is also an important added value." |
| Limitations of one-on-one peer support | Matching process challenges | Different treatment pathways | "We were a great match, especially in the beginning. We both had triple negative breast cancers, so our chemo therapy was the same. But then our ways split, because we did not have the same operation. And we were still in contact with each other, but you can no longer share experiences. And especially that feeling when: 'Oh, well that happened to you too?' gives the feeling that you can support each other." |
|  |  | Same treatment, but no social match | “I had so many questions about the DIEP reconstruction, and that was the connection. But the match was only based on the treatment, there was no social match.” |
|  |  | Conversation dies | "She called me once and we physically met, but afterwards the conversation died." |
|  | Dealing with responsibility | A buddy is not a professional | “I felt one of my buddies was a bit in a crisis at one point. And that was also in COVID-19 times. Her operation got postponed and she got really devastated. Then I contacted X (Buddy House initiator) and she contacted the hospital. But at that point I realized, I'm not a therapist. So what do you do when someone's really devastated?” |
|  | Lack of clear expectations | Unclear what to expect of a Buddy | “How do you know what to expect? As well for being as for having a buddy.” |
|  |  | Wrong expectations | "I have the feeling that she expects me to be a new friend to share everything with, but I would like to frame this relationship a bit more." |
|  |  |  | "I thought I was being connected to a poor soul to help her out. But it all turned out different than I had in mind." |
| Wishes | Evaluation moment | Providing feedback to the Buddy House | "When you are connected with someone, but that persons' story touches you in a way that it could possibly get you down, an option for intervision should be available." |
|  |  |  | “When a buddy tells something that worries or touches you, there should be a possibility for intervision.” |
|  | Visible profile | Visible characteristics | "Having something like a mini passport, showing your age, whether you have kids, which treatment you get." |
|  |  | Visible communication preferences | "Maybe provide some tips, like; make sure the first time you talk, make agreements on what you both want. What do you expect? How often and how intensively?" |
|  |  | Visible patient needs | "Maybe it sounds a bit plastic, but maybe it is an idea to show patients' needs. What is the reason you're connected to someone. For one it is to have a person to share their story, while for someone else it is more about practical questions about the operation." |
| *Abbreviations*: COVID-19 Coronavirus disease 2019; DIEP deep inferior epigastric artery perforator. | | | |

**Appendices**

**APPENDIX I**: Reflexivity

Since researchers bring their own backgrounds to the phenomenological approach, practicing reflexivity is critical. Therefore, it is important to provide the following contextual information: the lead author (BAMJ) has a background in medicine, currently working on her thesis on oncological breast surgery and has no relationship with participants. Her collaborators represent various backgrounds including epidemiology (HMV, DYA), Plastic and Reconstructive Surgery (CAB, DYA), Oncological Surgery (AD, ELP), medicine (TLD) and members of the board of the Breast Cancer Buddy House (AD, CAB). The different backgrounds reflected in various perspectives on the research question and all researchers were involved in the analytic process.

**APPENDIX II:** Beneficial initiatives specific for the Buddy House program

*"Also the walk-ins were really helpful, because to me it was like; You have this imagination of having cancer, mainly old people. Then I saw buddy C sitting there, and all those other ladies who survived breast cancer. That is also an important added value.”*

Initiatives such as the organized walking groups, monthly webinars and regular walk-ins are a great added value to peer support according to participants. The walking groups offer a pleasant manner of communication, as it is easier to talk to someone you don’t really know while walking instead of looking each other right in the eyes. The walks are organized in groups, which brings the opportunity to meet other breast cancer patients or survivors and make new buddy connections according to personal needs and preferences. The theme evenings are experienced as very informative with a diversity of breast cancer related subjects, and create the opportunity to make new connections.
